# The AI Colleague: Reimagining Interprofessional Collaboration and Education in Medicine

**DOI:** 10.2196/94034

**Published:** 2026-08-14

**Authors:** Chisung Yuh, Chul-Hyun Cho, Young-Mee Lee

**Affiliations:** 1 Department of Medical Education, Korea University College of Medicine, Seoul, Republic of Korea; 2 Department of Psychiatry, Biomedical Informatics, and Medical Education, Korea University College of Medicine, 73 Goryeodae-ro, Seongbuk-guSeoul, 02841, Republic of Korea, 82 2 920 5505

**Keywords:** AI, interprofessional education, interprofessional collaboration, medical education, competency-based education

## Abstract

AI is no longer confined to optional decision support; it is becoming a routine presence in clinical workflows, shaping diagnostic hypotheses, triage priorities, risk estimates, and documentation. Yet most educational responses still treat AI as a tool operated by an individual clinician. This framing underestimates how AI reshapes the actual unit of practice: the interprofessional team. We propose the concept of AI-expanded interprofessional collaboration (AI-IPC), in which AI systems function as consequential participants in team cognition—not as moral agents, but as sources of recommendations, uncertainty, and constraints that reorganize communication, authority gradients, and accountability. Building on interprofessional education (IPE) theory, situated learning, and distributed cognition, we argue that “AI literacy” alone is insufficient; learners must be trained to coordinate human-AI-human collaboration in realistic clinical settings. We outline a pragmatic, theory-aligned approach for AI-expanded interprofessional education (AI-IPE): clarifying boundary conditions for AI participation, mapping AI-specific subcompetencies onto established IPE frameworks, and evaluating performance at the level of team behaviors rather than knowledge recall. We present the Interprofessional Education Collaborative (IPEC)–aligned evaluation scaffold with concrete learning activities and assessment approaches, and we outline how the approach can be tailored across undergraduate, postgraduate, and continuing education settings. We further emphasize that implementation requires digital infrastructure, institutional governance, and educator capacity that bridges AI, clinical workflow design, and IPE facilitation. Finally, we address the deliberate use of the “AI colleague” metaphor—not to anthropomorphize AI, but to make the relational and coordinative demands of AI integration visible and teachable. The pedagogical aim is calibrated, critical engagement: teams that verify, question, and, when warranted, override AI contributions rather than defer to them. AI will not replace interprofessional collaboration; it will change what collaboration requires. Education should make that change explicit, rehearsable, and assessable.

## Introduction: The Unit of Practice Is Shifting

In contemporary clinical settings, AI is increasingly present at the point where decisions are made: in radiology workflows, risk prediction and early warning dashboards, medication safety alerts, ambient documentation systems that draft clinical notes from recorded encounters [1], and emerging “agentic” systems that can coordinate tasks across clinical and operational domains [2-5]. Even when AI is not autonomous, its outputs can become the scaffolding around which teams organize attention, deliberation, and documentation.

Medical education has responded with calls for AI literacy, data science training, and ethical competencies [6-8]. These are necessary—but they are also incomplete. Many educational proposals implicitly assume that the unit of practice is a single clinician operating a tool. In reality, the unit of practice in modern health care is often the team. Interprofessional collaboration (IPC) is not merely a social ideal; it is a cognitive and organizational strategy for managing complexity, uncertainty, and accountability in patient care [9,10]. The World Health Organization defines collaborative practice as occurring when “multiple health workers from different professional backgrounds work together with patients, families, carers, and communities to deliver the highest quality of care” [11]. In everyday medicine, IPC takes concrete forms: a ward round in which physicians, nurses, and pharmacists reconcile a medication plan; a tumor board in which radiologists, pathologists, surgeons, and oncologists negotiate a staging decision; a rapid response team in which nursing observations trigger escalation and shared decision-making. Each of these settings is now a plausible site of AI participation—an early warning score prompting the escalation, a draft imaging report anchoring the tumor board discussion, an interaction alert reshaping the medication review.

Recent literature has begun to map the intersection of AI and interprofessional education (IPE). A mixed methods scoping review identified 15 studies exploring AI applications aimed at fostering interprofessional teamwork, including virtual reality simulations, clinical decision support systems, and machine learning algorithms [12]. Similarly, simulation-based approaches leveraging big data analytics and AI have been proposed to enhance IPC training [13]. However, most of this work addresses AI as a tool for improving existing IPE or describes what AI applications currently exist. What remains underexplored is a conceptual and practical framework for how IPE itself must be redesigned when AI becomes a routine participant in clinical team deliberations—and how such redesigned IPE can be assessed.

This viewpoint addresses that gap. Starting from a simple observation—that AI is beginning to reconfigure collaboration—we propose a framework for AI-expanded IPE (AI-IPE) that focuses on the educational design and evaluation of team competencies under AI-integrated conditions. Our contribution is not to catalog AI applications for IPE, but to articulate what teams must learn to do differently when AI outputs enter everyday clinical workflows, and how that learning can be structured, practiced, and assessed. We write primarily for health professions educators, curriculum and assessment leaders, and directors of IPE programs; clinical supervisors, institutional leaders responsible for AI governance, and health professions education researchers are secondary audiences with clear stakes in the argument. Two commitments frame everything that follows. The first concerns our aim: we want to cultivate disciplined skepticism as a collaborative habit, so that teams verify AI outputs, question them openly, and override them when the evidence warrants, rather than defer to them. The second concerns what we do and do not claim about AI: we do not suggest that AI systems think, understand, or bear responsibility. The educational problem is precisely that systems without understanding now produce outputs that teams must treat as consequential.

## Guiding Educational Frameworks

Because the argument draws on several educational traditions, we introduce them at the outset. Situated learning theory holds that competence develops through legitimate peripheral participation in authentic activity: people learn to collaborate by collaborating, within communities of practice rather than in lecture halls [14,15]. Distributed cognition extends the unit of analysis beyond the individual mind: cognitive work in clinical settings is distributed across people, artifacts, and environments, so a risk score or a generated report is not merely information but a structural participant in how a team thinks [16,17].

Competency-based medical education (CBME), the organizing paradigm of contemporary training, completes the frame: it defines outcomes as observable behaviors and asks what learners can be entrusted to do in real practice [18,19]. For interprofessional competencies specifically, the Interprofessional Education Collaborative (IPEC) Core Competencies (version 3) provide the most widely used anchor, organizing collaboration into values and ethics, roles and responsibilities, interprofessional communication, and teams and teamwork [20]. These perspectives matter here because each is stressed differently when AI enters the room: situated learning tells us where AI collaboration must be learned, distributed cognition tells us why AI outputs reshape team reasoning, and CBME and IPEC tell us how the resulting competencies can be specified and assessed. We return to each throughout.

## From “Tool” to “Colleague”: A Deliberate Conceptual Choice

Why insist on the language of “colleague”? Because it forces education to confront a neglected dimension of AI integration: interaction. In many clinical deployments, AI is not just an instrument that produces a number; it enters conversations—explicitly or implicitly—as something that can be cited, deferred to, or overridden. It can shift authority gradients (“the algorithm says...”), narrow the hypothesis space, and alter the timing of action.

Consider a concrete example. Machine learning–based sepsis early warning systems are now in routine clinical use. The TREWS (Targeted Real-Time Early Warning System) system, implemented across 5 hospitals in the Johns Hopkins Health System, generates real-time sepsis alerts that clinicians review and confirm [21], and the COMPOSER deep learning model, deployed in emergency departments at UC San Diego Health, continuously monitors more than 150 patient variables and issues a nurse-facing alert when sepsis risk is elevated [22]. In both deployments, the alert does not simply produce a number; it triggers a communication sequence—the nurse evaluates the alert, contacts the physician, and together they decide whether to initiate a treatment bundle. Prospective evaluations have reported lower mortality and faster treatment when this human-in-the-loop workflow functions well [21,22]. Yet parallel evidence shows that alert fatigue, mistrust, and poorly calibrated reliance can undermine outcomes [23]. These failure modes are not technical—they are social-cognitive: they emerge in teams, under time pressure, with uneven expertise, and in the presence of accountability demands.

A fair question is why AI deserves special educational attention when clinical teams already work amid infusion pumps, physiological monitors, and rule-based decision support systems that interpret data and issue recommendations. We see continuity rather than rupture: automation bias and alarm fatigue were documented long before modern machine learning [24]. Three features of contemporary AI nonetheless change the collaborative stakes. First, these systems are adaptive and often opaque; their behavior shifts with training data and context in ways that resist the simple mental models clinicians build for conventional devices. Second, generative systems produce fluent narratives—draft reports, summaries, and replies—that enter team communication in the same register as human speech rather than as a beep or a threshold flag. Third, AI now spans the entire workflow, from triage to documentation, so its outputs cross professional boundaries instead of remaining within a single device-operator dyad. We agree that regulatory clarity—ensuring that AI tools are appropriately categorized and governed as medical devices—is a necessary complement to education [25,26]; our claim is that education must additionally prepare teams for the interactional consequences that follow even when such governance is sound.

We are aware that the “colleague” label invites criticism. AI is not a moral agent, does not hold professional licensure, and cannot bear accountability. Anthropomorphism can obscure power asymmetries and deflect responsibility. There is also a subtler hazard, which deserves emphasis at the outset rather than as a closing footnote: clinicians already tend to experience fluent AI systems as thinking interlocutors when they are in fact probabilistic models that do not understand the propositions they generate. Any pedagogy that borrows the language of collegiality must build in this corrective at the same time. We use the term deliberately, not to grant AI personhood, but as a pedagogical provocation: if education treats AI as merely another stethoscope, it will fail to prepare learners for the relational complexity that AI actually introduces. The “colleague” metaphor is a heuristic that makes the coordinative demands of AI integration visible and discussable in educational settings. Throughout this paper, we use the more precise term “consequential participant” to maintain conceptual rigor while retaining the pedagogical utility of the metaphor. The metaphor earns its keep only if it makes AI easier to question, not easier to trust.

From the perspective of distributed cognition theory, this reframing is well grounded. As noted earlier, cognition is spread across people, artifacts, and environments rather than being sealed inside individual minds [16,17]. In clinical settings, AI outputs function as cognitive artifacts that shape how teams allocate attention, interpret uncertainty, and coordinate action. When an AI system generates a risk score that restructures team communication, it is participating in the distributed cognitive system—regardless of whether we call it a colleague or a tool.

Automation bias—overreliance on computer suggestions even when incorrect—has been documented extensively in clinical decision support, and it can be amplified when AI outputs carry perceived objectivity or institutional endorsement [24,27]. Equally, unreflective rejection of AI can occur when trust is poorly calibrated, leading to missed opportunities for safety and efficiency. Both failure modes are social-cognitive challenges that demand collaborative—not merely individual—educational responses. IPE already possesses relevant tools for this problem. The TeamSTEPPS (Team Strategies and Tools to Enhance Performance and Patient Safety) framework, developed by the Agency for Healthcare Research and Quality (AHRQ), provides training in structured mutual challenge; its “two-challenge rule” obliges any team member to voice a safety concern at least twice and to escalate if the concern is not acknowledged [28]. AI-expanded interprofessional collaboration (AI-IPC) extends exactly this repertoire to a nonhuman participant: teams need norms that make questioning an algorithmic output as routine—and as psychologically safe—as questioning a senior colleague’s plan. Framed this way, the competency at stake is not to treat AI as “another professional” but to ensure that its suggestions receive the same disciplined scrutiny that well-functioning teams apply to any consequential claim.

## Defining AI-IPC

AI-IPC is not intended as a metaphor. It is a structural description of how team-based care changes when AI outputs are embedded in workflows. We define AI-IPC as follows: a mode of interprofessional practice in which AI systems function as consequential participants in team deliberation by generating recommendations, risk estimates, or synthesized narratives that materially influence attention, reasoning, and documentation. Professional accountability remains entirely human; AI is a participant in the sense that it contributes inputs that teams must negotiate.

AI becomes a meaningful participant when 3 boundary conditions are satisfied:

- Operational integration—AI outputs are routinely available at the point of care (eg, triage dashboards, imaging report generation, and ambient note drafting).
- Override governance—there are explicit norms or policies for accepting, contesting, or overriding AI recommendations.
- Traceable accountability—teams must document and justify decisions, including when they depart from AI output.

**Figure 1.**
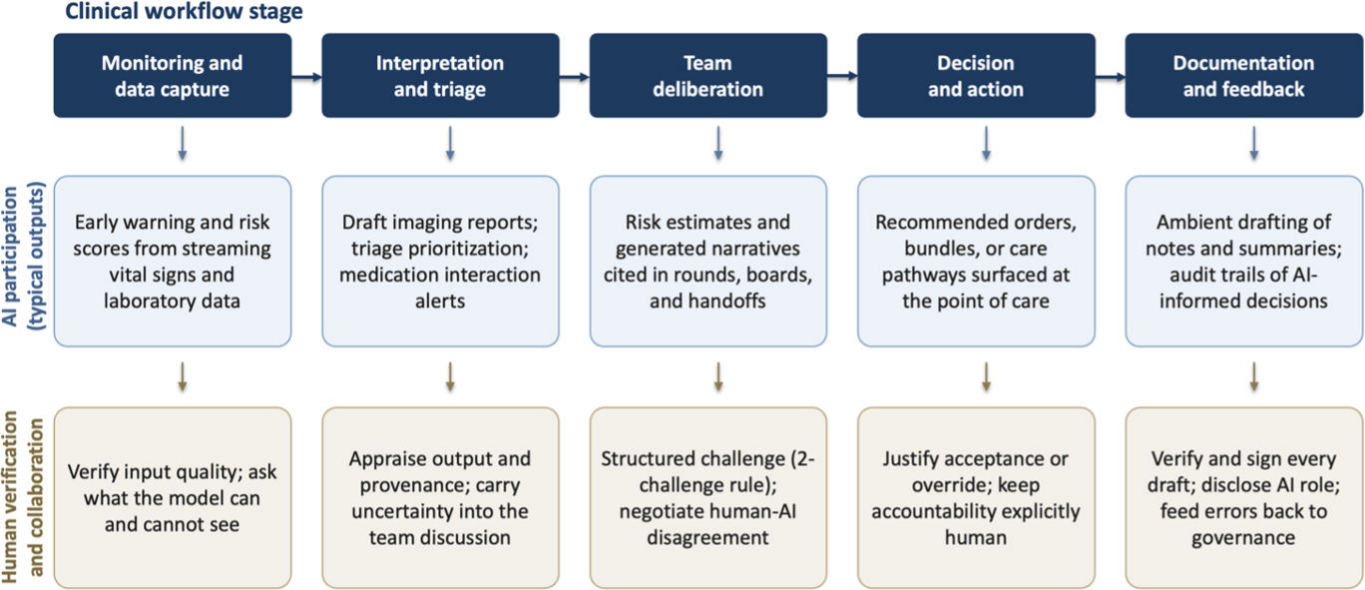
Points of AI participation across a generic clinical workflow, and the corresponding human verification and collaboration practices that AI-expanded interprofessional education (AI-IPE) must rehearse. Accountability remains human at every stage: AI contributes inputs that teams verify, question, and—when warranted—override.

These conditions are increasingly common as multimodal generative systems mature and as “agentic AI teammates” are envisioned for coordinated task execution [4,29]. The question for education is not whether AI will be present, but whether clinicians will be prepared to collaborate safely and transparently in AI-IPC environments. Figure 1 locates these boundary conditions in a generic clinical workflow: it marks where AI outputs typically enter team activity—from monitoring and triage through deliberation, decision, and documentation—and pairs each entry point with the human verification and collaboration practices that education must make rehearsable.

## Why AI Literacy Is Necessary but Not Sufficient

As used here, AI literacy denotes the cluster of knowledge and skills that allows health professionals to understand what AI systems can and cannot do, interpret their outputs, and recognize their ethical and legal implications—including data provenance, algorithmic bias, and privacy [6-8]. AI literacy typically focuses on understanding model capabilities, limitations, and ethical issues. These are essential foundations. However, they do not specify how teams should work when AI outputs collide with human judgment, when uncertainty must be communicated across disciplines, or when documentation must assign responsibility for an AI-influenced decision. Such moments are not rare edge cases—they are the routine friction points of AI integration. Ambient documentation is a case in point: generated drafts are fluent and mostly accurate, which is precisely why they demand line-by-line verification before signature. Knowing in the abstract that errors occur does not settle who verifies, who signs, and how a team recovers when a fabricated detail is discovered after the fact [1].

Consider a common team scenario: a nurse reports an AI-based early warning score suggesting deterioration, while a resident believes the patient is stable. A pharmacist raises concerns about a medication recommendation surfaced by an AI-driven interaction checker. A senior physician asks whether the AI output is explainable enough to justify an invasive intervention. Each question is simultaneously clinical and collaborative: it requires negotiation across roles, explicit communication of uncertainty, and a clear account of who decides and who documents. These are IPE competencies—but with an AI-specific twist.

In other words, the educational target is not “how to use AI,” but “how to collaborate when AI is in the room.” Part of that competence is provenance awareness: registering that a given artifact is AI-crafted and adjusting practice accordingly. Provenance matters for at least 4 reasons. It signals how an output was produced—statistical association rather than clinical reasoning—and therefore how it is likely to fail. It determines what verification is owed before the output informs action. It shapes documentation and disclosure, including what patients are told about how a recommendation arose. And it calibrates the epistemic weight a team should grant the output in discussion, especially when it conflicts with human judgment. A team that cannot tell—or does not ask—whether a narrative was machine-generated can perform none of these adjustments.

## Situated Learning and Distributed Cognition: Making AI-IPC Teachably Real

IPE succeeds when it is not purely declarative but practiced in contexts that resemble real work. As outlined earlier, situated learning locates competence in social participation and authentic activity [14,15]. IPE, at its best, is a situated apprenticeship in collaboration.

AI-IPC strengthens the case for situated approaches. AI outputs are context-sensitive: the same recommendation can be interpreted differently depending on role, workflow, patient context, and institutional norms. Moreover, AI introduces new “objects” of coordination—probabilistic risk scores, generated narratives, uncertainty estimates, and provenance questions—that must be discussed in real time. These are not learned effectively through lectures alone. Generative systems can themselves supply raw material for such training: large language models can draft clinical cases with realistic parameters, although comparative work in dermatology education found that AI-generated cases, while rich in descriptive detail, lacked some of the clinical depth and dilemma quality of instructor-crafted ones—a useful reminder that educational artifacts generated by AI require the same critical appraisal as clinical outputs [30].

Distributed cognition theory further illuminates why simulation is essential. Because cognitive work in AI-IPC environments is distributed across clinicians, AI artifacts, and documentation systems, effective team performance requires rehearsing the entire sociotechnical system—not merely individual AI skills [17]. Learners must practice interpreting AI outputs while simultaneously communicating with team members, managing uncertainty, and documenting decisions—activities that only emerge in realistic interprofessional settings.

Thus, we argue for AI-IPE: interprofessional education that explicitly incorporates AI as a structured participant in the learning environment so that learners develop collaborative fluency under AI-integrated conditions.

## AI-IPE: Extending, Not Replacing, Established Frameworks

AI-IPE does not replace what we already know about IPE. Instead, it should extend established frameworks with AI-specific subcompetencies. The IPEC Core Competencies (version 3), introduced above, remain the natural anchor: values and ethics, roles and responsibilities, interprofessional communication, and teams and teamwork [20]. Our contribution is to make explicit how each domain is reshaped when AI outputs enter into everyday team deliberations.

For example, values and ethics now include competence in recognizing algorithmic bias, understanding transparency limits, and protecting patient autonomy in AI-informed decisions [26,31]. Roles and responsibilities require explicit agreements about who interprets AI outputs, who validates them, and how accountability is documented. Interprofessional communication must incorporate communication of probabilistic uncertainty and provenance [32]. Teams and teamwork must manage disagreement—among professionals and between professionals and AI outputs—without defaulting to either algorithmic deference or reflexive dismissal.

CBME further supports this direction, as introduced earlier, because AI-IPE competencies are observable behaviors: critical appraisal of AI outputs, justification of override decisions, and transparent documentation [18,19]. The educational question becomes: what can learners be entrusted to do—safely, ethically, and collaboratively—when AI is part of the workflow?

Table 1 contrasts traditional IPE with AI-IPE across 5 dimensions, emphasizing that the shift is not merely technical but relational.

**Table 1.** Traditional interprofessional education (IPE) and AI-Expanded IPE (AI-IPE) compared across 5 dimensions.

Dimension	Traditional IPE	AI-IPE	Educational implication
Team composition	Human professionals only	Human professionals+AI systems producing consequential inputs	Teach human-AI-human collaboration
Core coordination problem	Role clarity and communication across professions	Role clarity+AI interpretation, trust calibration, and override governance	Train teams to negotiate AI output
Uncertainty management	Clinical uncertainty shared across humans	Clinical uncertainty+algorithmic uncertainty and provenance	Practice communicating probabilistic outputs
Accountability	Human accountability, professional norms	Human accountability with AI-influenced decisions and audit trails	Train documentation and justification practices
Learning setting	Simulation of interprofessional scenarios	Simulation including AI-generated recommendations, conflicts, and error modes	Situated rehearsal of AI-IPC^a^

a IPC: interprofessional collaboration.

## Tailoring AI-IPE Across the Learner Continuum

AI-IPE cannot be one-size-fits-all, because awareness of and familiarity with AI—and the authority to act on its outputs—differ systematically across the educational continuum. At the undergraduate (prelicensure) stage, learners are still forming their professional identities and arrive with heterogeneous levels of AI exposure, much of it from consumer tools rather than clinical systems. Here, the emphasis should be on foundational AI literacy, a shared interprofessional vocabulary for AI outputs, and low-stakes simulation in which questioning an algorithm is practiced before questioning carries real consequences. Mixed-profession groups are valuable early, precisely because norms of cross-professional challenge are easiest to establish before hierarchies harden.

In postgraduate training, learners hold real decision authority within specialty-specific workflows, and AI-IPE should correspondingly move into workplace-based rehearsal of override decisions, escalation pathways, and documentation practices, exercised in the learner’s actual clinical context, with supervisors assessing entrustment under AI-integrated conditions. For continuing professional development, the audience is intact teams with entrenched routines and highly variable AI familiarity—including inverted expertise gradients in which junior members understand the AI better than their seniors. Short, team-based rehearsals embedded in existing quality and safety structures (eg, morbidity and mortality conferences or periodic simulation refreshers) fit this group better than standalone coursework. Facilitation should be planned as deliberately as content: pairing an experienced IPE facilitator with a clinical informatician covers both the group dynamics and the technical substance, and the composition of each learner group—homogeneous or mixed across professions and career stages—should be an explicit design decision rather than an accident of scheduling.

## From Concept to Assessment: A Proposed, IPEC-Aligned Evaluation Scaffold

Because AI-IPE targets team behaviors, evaluation must focus on performance in collaboration—not solely on knowledge. We therefore propose (not prescribe) an evaluation scaffold aligned with IPEC domains and CBME principles, designed to assess learning and behavior levels (Kirkpatrick levels 2‐3; Table 2) [9,33]. Textbox 1 complements the scaffold with brief cases and reflective questions that educators can use to seed discussion at any of these levels.

**Table 2.** A proposed Interprofessional Education Collaborative (IPEC)–aligned evaluation framework for AI-expanded interprofessional education (AI-IPE)^a^.

IPEC domain	AI subcompetency	Learning activity	Assessment approach	Level
Values and ethics	Recognize bias, transparency limits, and patient autonomy issues in AI-informed decisions	AI-assisted triage discussion with fairness challenge (eg, biased risk score across demographics)	Bias-identification rubric+structured reflection	Learning
Roles and responsibilities	Clarify accountability for interpreting, validating, and documenting AI output	Interprofessional simulation where AI recommendation conflicts with clinician judgment (eg, sepsis alert vs clinical assessment)	Justification checklist+documentation audit	Learning or behavior
Interprofessional communication	Communicate probabilistic AI output (uncertainty/provenance) to team and patient	Standardized patient encounter explaining AI-informed plan (eg, explaining AI-generated risk score to patient and family)	OSCE^b^-style communication rating+debrief transcript review	Learning or behavior
Teams and teamwork	Manage disagreement constructively; calibrate trust and coordinate overrides	Multidisciplinary case conference with AI-generated report and embedded error mode (eg, hallucinated findings)	Team performance rubric+override-decision log review	Learning or behavior

a This table is proposed as an illustrative scaffold. Institutions should adapt it to local contexts, available AI tools, and governance requirements. Activities labeled “Learning or Behavior” assess Kirkpatrick level 2 when conducted as simulation, and level 3 only when they involve direct observation of behavior in live clinical practice (eg, workplace-based observation, override-decision audits, and multisource feedback).

b OSCE: objective structured clinical examination.

Textbox 1. **Illustrative AI-IPC cases with reflective questions for educators.**

Case 1: The contested alert. At 2 AM, an early warning system flags a postoperative patient for probable sepsis. The bedside nurse finds the patient sleeping comfortably; the covering resident, examining the patient, is reassured and inclined to dismiss the alert. The nurse, following unit policy, asks the resident to state a rationale for the override so it can be documented.

Reflective questions: What would count as an adequate justification to override this alert? Who documents the override, and where? If the patient deteriorates 3 hours later, how will the team’s earlier reasoning be reconstructed—and defended?

Case 2: The fluent draft. An ambient documentation system produces a discharge summary that reads well but attributes a normal neurological examination that no one performed. The intern notices the discrepancy only because a physiotherapist queries the mobility plan that was built on it.

Reflective questions: Who is responsible for verifying and signing AI-drafted documentation? How should the team repair the record—and the plan—once a fabricated detail has propagated? What should the patient be told?

Case 3: The skewed score. During discharge planning rounds, a nurse notices that a readmission risk score consistently rates patients from one demographic group as lower risk, with the effect that follow-up resources are being allocated away from them.

Reflective questions: How should the nurse raise this concern, and to whom? What does a 2-challenge escalation—TeamSTEPPS’s [Team Strategies and Tools to Enhance Performance and Patient Safety] expectation that a safety concern be voiced at least twice before escalating—look like when the “colleague” being challenged is an algorithm, and the humans in the room did not build it? At what point does an educational observation become a governance report?

## What Makes AI-IPE Feasible: Infrastructure, Governance, and Educator Capacity

A concept only matters educationally if institutions can actually implement and assess it. AI-IPE, therefore, depends on 3 enabling conditions that are often underestimated.

The first condition is digital infrastructure. AI-IPE requires learning environments where AI outputs are not hypothetical but operationally similar to what teams will encounter. This can include AI-enabled simulation cases (eg, generated imaging reports or risk dashboards), structured error-mode scenarios (eg, biased outputs or hallucinated narratives), and digital capture of decisions [34,35]. Assessment also requires digitized tools that can record override decisions, documentation patterns, and team interactions (eg, audit trails, rubric platforms, or annotated transcripts). Without such tools, evaluation collapses into self-reporting and loses credibility.

The second condition is institutional governance. Trustworthy AI guidance emphasizes transparency, safety, and accountability as prerequisites for clinical deployment [25,26]. In education, these translate into teachable rules: documentation norms for AI-informed decisions, escalation pathways when AI output is contested, and structured communication with patients about the role of AI in their care. Clinical AI reporting frameworks (eg, CONSORT-AI [Consolidated Standards of Reporting Trials–AI Extension], SPIRIT-AI [Standard Protocol Items: Recommendations for Interventional Trials–AI Extension], and DECIDE-AI [Developmental and Exploratory Clinical Investigations of Decision support systems driven by AI]) provide models for how transparency and accountability can be operationalized [36,37]. Training without governance risks normalizing unsafe practices; governance without training risks creating policies that are ignored in practice.

The third condition is educator capacity. AI-IPE cannot be delivered by AI experts alone, nor by IPE facilitators without AI literacy. Educators must hold an integrated understanding of (1) AI capabilities and limitations, (2) IPE or IPC theory and facilitation, (3) clinical workflow realities, and (4) digital assessment methodologies. Faculty development should, therefore, be an explicit component of AI-IPE implementation [7,8]. In practice, institutions may need interdisciplinary educator teams (clinical faculty, medical education specialists, informaticians, and quality or safety leaders) to co-design AI-IPE cases and assessments. If educators cannot model how to interrogate AI output, negotiate disagreement, and document accountability, learners will be trained to perform either deference or skepticism—rather than calibrated, collaborative judgment.

## Practical Next Steps for Implementation

For institutions persuaded by the argument so far, the practical question is where to begin. A staged sequence keeps the work tractable and lets faculty capacity grow alongside it.

1. Map the AI already present. Audit where AI outputs actually enter local workflows—triage scores, imaging drafts, interaction alerts, ambient notes—and identify the 2 or 3 that most often cross professional boundaries.
2. Convene an interdisciplinary design team. Pair IPE facilitators with clinical informaticians, frontline clinicians from each profession, and quality and safety leads; none of these groups can design AI-IPE alone.
3. Retrofit one existing IPE scenario. Rather than building a new curriculum, add a realistic AI artifact—with an embedded error mode—to a scenario the institution already runs, and script the decision points it creates.
4. Pilot with an explicit debriefing protocol. Run the scenario with mixed-professional groups and debrief on trust calibration, challenging behavior, and documentation—not only on clinical correctness.
5. Attach the assessment early. Apply an adapted version of the Table 2 scaffold, with digital capture of override decisions and team communication, so that evaluation data accumulate from the first iteration.
6. Report through governance channels and iterate. Feed findings back to the institutional AI governance structure, align teaching with local policies, and expand into additional scenarios and learner groups as faculty capacity grows.

## Implications for Patients

The case for AI-IPE is ultimately a case about patient care. Collaborative practice, by the definition adopted above, includes patients, families, and carers—not only professionals [11]. When teams rehearse uncertainty communication and provenance disclosure, the intended beneficiary is the patient who must decide whether to accept a plan that an algorithm helped to shape. Focus-group research indicates that patients bring specific and reasonable concerns to AI-informed care—safety, threats to choice, cost, data-source bias, and data security—and that their acceptance is contingent on seeing these concerns addressed [38]. A team trained to explain what an AI contribution was, and was not, is better positioned to support informed consent and shared decision-making than a team that either conceals the algorithm or hides behind it. We treat the fuller patient perspective—including whether AI-IPE measurably improves patient understanding and trust—as an empirical question for the research agenda below.

## Limitations and Research Agenda

This viewpoint starts from a conceptual claim: AI reshapes the unit of collaborative practice and therefore reshapes IPE. This claim should be open to empirical scrutiny. Future work should examine whether AI-IPE improves team performance in domains such as the quality of override decisions, the clarity of uncertainty communication, documentation transparency, and patient understanding. Designs might include simulation-based controlled studies, longitudinal workplace assessments, and implementation evaluations aligned with reporting frameworks for clinical AI (eg, CONSORT-AI, SPIRIT-AI, and DECIDE-AI) [36,37]: syntheses of the resulting evidence should in turn follow emerging standards for AI systematic reviews [39]. Because the present argument is conceptual and narrative in nature, a natural next step is a domain-specific implementation study. Sepsis response teams and oncology multidisciplinary conferences are attractive first testbeds: AI tools are already consequential in both, and the team decision points are well demarcated. Such a study would operationalize the Table 2 scaffold in a single clinical domain, with prespecified team-level outcomes before any claim of generality is made.

A further limitation is heterogeneity: AI systems differ, workflows differ, and cultures of collaboration differ. AI-IPE should therefore be conceptualized as a framework for local adaptation rather than a single standardized curriculum. Additionally, our proposed evaluation scaffold (Table 2) is illustrative; empirical validation across institutions and professional contexts is needed before it can serve as a prescriptive assessment standard.

Finally, we acknowledge that the “AI colleague” metaphor has limits, as we have emphasized from the outset. It risks conveying agency, intentionality, or accountability where none exists. The value of the metaphor lies precisely in its provocation: by framing AI as a colleague, we make visible the coordination demands that would otherwise remain hidden under the label of “tool.” The purpose is to prompt educational action, not to make ontological claims about AI.

## Conclusion: Education Should Make Collaboration With AI Teachable

AI will not replace interprofessional collaboration; it will change what collaboration requires. When AI outputs become consequential inputs into team cognition, the collaborative work of health care teams shifts: communication, trust calibration, disagreement management, and documentation become AI-conditioned practices. Treating AI as merely a tool is educationally insufficient because it hides the interactive, relational nature of AI integration.

AI-IPC and AI-IPE provide a language for making this shift visible, teachable, and testable. Grounded in situated learning and distributed cognition and aligned with established IPE frameworks, AI-IPE asks learners to rehearse human-AI-human collaboration under realistic conditions and to demonstrate observable team behaviors—verification, structured challenge, justified override, and transparent documentation—especially in moments of uncertainty and disagreement. Realizing this vision requires more than enthusiasm for AI: it requires infrastructure, digitized assessment, institutional governance, and educators equipped to bridge AI, workflows, and interprofessional pedagogy. If we can teach teams to work well with each other, we can also teach them to work well with AI—provided that we are explicit about what that work entails.

## Abbreviations

AHRQ: Agency for Healthcare Research and Quality
AI-IPC: AI-expanded interprofessional collaboration
AI-IPE: AI-expanded interprofessional education
CBME: competency-based medical education
CONSORT-AI: Consolidated Standards of Reporting Trials–AI Extension
DECIDE-AI: Developmental and Exploratory Clinical Investigations of Decision Support Systems Driven by AI
IPC: interprofessional collaboration
IPE: interprofessional education
IPEC: Interprofessional Education Collaborative
SPIRIT-AI: Standard Protocol Items: Recommendations for Interventional Trials–AI Extension
TeamSTEPPS: Team Strategies and Tools to Enhance Performance and Patient Safety
TREWS: Targeted Real-Time Early Warning System
